# CAT: computer aided triage improving upon the Bayes risk through *ε*-refusal triage rules

**DOI:** 10.1186/s12859-018-2503-9

**Published:** 2018-12-21

**Authors:** Nicolas Hengartner, Leticia Cuellar, Xiao-Cheng Wu, Georgia Tourassi, John Qiu, Blair Christian, Tanmoy Bhattacharya

**Affiliations:** 1 Los Alamos National Laboratory, PO Box 1663, Los Alamos, 87545 NM USA; 2 Louisiana State University, 2020 Gravier Street, 3rd Floor, New Orleans, 70112 LA USA; 3 Oak Ridge National Laboratory, PO Box 2008, Oak Ridge, 37831 TN USA

**Keywords:** Machine learning, Classification

## Abstract

**Background:**

Manual extraction of information from electronic pathology (epath) reports to populate the Surveillance, Epidemiology, and End Result (SEER) database is labor intensive. Systematizing the data extraction automatically using machine-learning (ML) and natural language processing (NLP) is desirable to reduce the human labor required to populate the SEER database and to improve the timeliness of the data. This enables scaling up registry efficiency and collection of new data elements. To ensure the integrity, quality, and continuity of the SEER data, the misclassification error of ML and NPL algorithms needs to be negligible. Current algorithms fail to achieve the precision of human experts who can bring additional information in their assessments. Differences in registry format and the desire to develop a common information extraction platform further complicate the ML/NLP tasks. The purpose of our study is to develop triage rules to partially automate registry workflow to improve the precision of the auto-extracted information.

**Results:**

This paper presents a mathematical framework to improve the precision of a classifier beyond that of the Bayes classifier by selectively classifying item that are most likely to be correct. This results in a triage rule that only classifies a subset of the item. We characterize the optimal triage rule and demonstrate its usefulness in the problem of classifying cancer site from electronic pathology reports to achieve a desired precision.

**Conclusions:**

From the mathematical formalism, we propose a heuristic estimate for triage rule based on post-processing the soft-max output from standard machine learning algorithms. We show, in test cases, that the triage rule significantly improve the classification accuracy.

## Introduction

The Surveillance, Epidemiology, and End Result (SEER) collects and curates cancer patient data from electronic pathology (e-path) reports. Those data are used for population-based research on cancer trends to develop cancer treatment and screening policy recommendations. That information is extracted manually by experts, which is labor intensive. This limits the geographical coverage of the database, currently, about 30% of all cancer cases in the United States are included in the SEER database.

Automating the data extraction using Machine Learning (ML) and Natural Language Processing (NLP) tools is desirable to reduce the human labor that is required to populate the SEER database, thereby increasing the geographical coverage of the database and improving the timeliness of the data. To insure the integrity, quality and general usefulness of that database, the classification algorithm needs to have a misclassification error that is no more than that of human experts. This is challenging because these experts supplement the pathology reports with extra information not always available to the machine learning tools. Thus, it is implausible that, even under the best circumstances, the ML/NLP algorithms can achieve the same small error rate achieved by human experts. Differences in registry format and the desire to develop a common information extraction platform further complicate the ML/NLP tasks.

The ML/NLP task of extracting information from e-path reports can be cast as a multi-class classification problem. The misclassification error of any machine learning classification algorithm is bounded from below by the Bayes risk [1]. In some instances, that lower bound may exceed the accuracy needed by the end user, thereby making machine learning not useful.

The classification depends on both the content and the context of pathology report. This leads to heterogeneity in the degree of difficulty for classifying electronic pathology reports, with some pathology reports being easy to classify while others being harder. If one can identify which pathology reports are easy/hard to classify, i.e., have small/large expected misclassification error, one may improve upon the Bayes error by only classifying automatically the reports that have small expected misclassification error, leaving to the experts the task of classifying the more challenging ones.

We call *triage* machine learning algorithms that selectively classify item. We show in this paper that optimal triage classification rules achieve misclassification error that are lower than the Bayes classifier, at the cost of not classifying a fraction of the items. The lower misclassification rate arises because we do not get penalized for refusing to classify, since these reports will be evaluated by experts. By strategically refusing not to classify a large fraction of the items, we have the opportunity to achieve arbitrarily small misclassification rate. But there are resource constraints that limit the fraction of reports we refuse to classify. For example, ignoring the issue of building an appropriate infrastructure, if we wanted the SEER database to cover 100*%* of population the USA with the same manpower as now, we could refuse to evaluate at most 30% of the reports.

In this paper, we present the mathematical foundation for Computer Aided Triage (CAT) by showing how it expands on existing concepts of statistical machine learning. The paper is structured as follows: we first formalize the notation and define mathematically the *ε*-triage rule. We, then, prove that an *ε*-triage rule has monotonic decreasing classification error with increasing refusal fraction *ε* and characterize the optimal “Bayes” *ε*-triage rule, and we relate it to the classical Bayes rule. In the next Section, we apply heuristics derived from these results to post-process deep learning classification of cancerous tumor sites from electronic pathology reports to achieve a desired level of confidence. In a fourth section, we provide another application of this methodology to risk management. Finally, the interested reader will find the proofs in the appendix.

## Mathematical formulation

### Preliminaries

In this paper, we make use of the following notation. Denote by {(*X*_1_,*Y*_1_),…,(*X*_*n*_,*Y*_*n*_)}*n* independent identically distributed random vectors with joint distribution

$$\begin{array}{@{}rcl@{}} {\mathbb P}\left[X=dx,Y=a_{j}\right] &=& p\left(a_{j},x\right)dx = p\left(\left.a_{j}\right|x\right)f(x)dx. \end{array} $$

The covariates $X_{i} \in {\mathbb X} \subset {\mathbb R}^{p}$ while the response variable $Y_{i} \in {\mathcal A} = \{ a_{1},\ldots,a_{m} \}$ is one of *m* labels.

A classifier $\hat Y(x)$ is a function from the feature space ${\mathbb X}$ onto the set of labels {*a*_1_,…,*a*_*m*_}. To any classifier $\hat Y(x)$, we can associate a partition *A*_1_,…,*A*_*m*_ of the feature space ${\mathbb X}$, defined by $A_{k} = \left \{ x \in {\mathbb X} : \widehat Y(x) = a_{k} \right \}$. The partition of the Bayes classifier *Y*^⋆^(*X*), which minimizes the misclassification error ${\mathbb P}\left [\widehat Y(X) \not = Y\right ]$ amount all measurable functions $\widehat Y(X)$, is given by

1 $$ A^{\star}_{k} = \left\{ x : p\left(a_{k}|x\right) \geq p\left(a_{j}| x\right), \quad j \not = k \right\},  $$

with the convention that if there exists two or more indexes for which we have equality, *x* is assigned to set with the lowest index to ensure that the sets $A_{k}^{\star }$ partition the feature space ${\mathbb X}$. See [1] for example.

### Optimal triage rule

While the Bayes error is a lower bound for the misclassification error, the optimal triage rule will have a lower misclassification error. Let us formally define the *ε*-*triage* rule.

#### **Definition 1**

A *ε*-triage is a function *T* from the feature space ${\mathbb X}$ into the extended set of labels {*a*_1_,…,*a*_*m*_}∪*∅*, where the label *∅* represents the “no classification” category, and

$${\mathbb P}[ T(X) = \emptyset ] \leq \varepsilon. $$

We can associate to a triage function $ \widehat {T}(x)$ the sets

$$B_{k} = \left\{ x \in {\mathbb X} : \widehat{T}(x) = a_{k} \right\}.$$

Unlike the sets *A*_1_,…,*A*_*m*_ defined for a classifier, the sets *B*_1_,…,*B*_*m*_ do not form a partition of ${\mathbb X}$. As a result, let us define the decision set $D = \cup _{k=1}^{m} B_{k}$, and the rejection set *D*^*c*^={*x*:*T*(*x*)=*∅*}.

For a triage rule, the misclassification error is only evaluated on the decision set *D*. That is, a refusal to classify does not get penalized. Formally, the loss function for a triage rule *T* is

2 $$  L(T) = {\mathbb P}[\! Y \not = T(X), X \in D].  $$

Note that the loss *L*(*T*) can be made arbitrarily small by making *D* small enough. To avoid this uninteresting answer, we constraint the size of the decision set *D* to satisfy

$${\mathbb P}[ \!X \in D] \geq 1-\varepsilon. $$

Our first theorem characterizes the minimizer of (2), thus providing an analogous result to Bayes rule in the classical classification context.

#### **Theorem 1**

The triage function *T*^⋆^ that minimizes $ {\mathbb P}[Y \not = T(X), X \in D]$ subject to ${\mathbb P}[X \in D] \geq 1-\varepsilon $, for a given 0<*ε*<1, is characterized by the sets

3 $$  D^{\star} = \left\{ x : \max_{j} p\left(\left.a_{j}\right|x\right) > b \right\}  $$

and

4 $$  B_{k}^{\star} = D^{\star} \cap \left\{ p\left(\left.a_{k} \right| x \right) \geq \max_{j \not = k} p\left(\left.a_{j}\right|x\right) \right\},  $$

where the parameter *b* is the smallest value such that

5 $$ {\mathbb P}[X \in D^{\star} ] \geq 1-\varepsilon.  $$

**Remark** Observe that the sets *D*^⋆^(*b*)⊂*D*^⋆^(*b*^′^) when *b*≥*b*^′^, or equivalently, when *ε*≤*ε*^′^. As a result, the loss of the optimal triage rule *L*(*T*^⋆^) is a monotone decreasing function in *ε*.

It is insightful to specialize Theorem 1 to characterize the optimal triage rule for binary labeled features.

#### **Corollary 1**

Suppose that *Y*∈{0,1} takes on only two values. Then the indecision region for the optimal triage rule is

6 $$ D^{c} = \left\{ x : \frac{1}{b} \leq \frac{p(1|x)}{p(0|x)} \leq b \right\},  $$

where *b* is the largest value such that ${\mathbb P}[X \in D] \geq 1- \varepsilon $.

A similar “indecision set”, based on the likelihood ratio, arises in sequential learning [2].

### Relationship to Bayes rule

It is instructive to relate the optimal triage rule to the Bayes rule. To this end, denote by *Y*^⋆^ the Bayes rule.

#### **Proposition 1**

The optimal triage rule is related to the Bayes classifier through the sets

$$B_{k}^{\star} = D^{\star} \cap A^{\star}_{k}. $$

The proof is immediate by comparing the sets (2) and (4). This description allows us also write the triage rule *T*^⋆^ in terms of the Bayes classifier *Y*^⋆^. To this end, define the function

$$Z^{\star}(x) = \left \{ \begin{array}{rr} 1 & x \in D^{\star} \\ \emptyset & x \not \in D^{\star} \end{array} \right., $$

with the convention that *a*_*k*_×*∅*=*∅*. The optimal triage rule can then be written as

7 $$  T^{\star} = Z^{\star} Y^{\star}.  $$

This allows us to reinterpret Theorem 1: The optimal triage rule is the Bayes rule on the restricted set where largest conditional probability max*jp*(*a*_*j*_|*x*)>*b*. That is, the optimal triage is Bayes rule provided that the conditional probability of the winning class is large enough. As a consequence, it is possible that a triage rule does never classifies a particular class if the conditional probability *p*(*a*_*k*_|*x*)<*b* for all *x*. Identification of such classes is instructive, as it identifies difficult classes. to classify.

## Heuristic: using soft-max to build a triage rule

The previous section describes how the optimal triage rule is a thresholding function of the (optimal) Bayes rule. In this section, we propose the heuristic triage estimator obtained by post-processing the soft-max produced by various machine learning algorithms. Looking at Eq. (7), we propose to use a classifier that produces a soft-max to define sets that mimmic the sets *D*^⋆^ and $B_{k}^{\star }$. If the soft-max output from a ML algorithm is a good estimate for some monotone increasing transformation of the actual conditional probabilities, then this heuristic will produce good triage rules. Other estimation strategies are possible, and will be explored in a future manuscript.

Formally, let ${\mathcal H}$ denote a collection of real-valued score functions *h*(*a*,*x*) of the class labels $a \in {\mathcal A}$ and features $x \in {\mathbb X}$. For any function $h \in {\mathcal H}$, the soft-max is defined by

$$q_{h}(a,x) = \frac{e^{h(a,x)}}{\sum_{b \in {\mathcal A}} e^{h(b,x)}}, $$

which can be used to define the classifier

$$\begin{array}{@{}rcl@{}} Y(x) &=& \arg \max_{k} h\left(a_{k},x\right)\\ &=& \arg \max_{k} q_{h}\left(a_{k},x\right). \end{array} $$

It is well understood while the soft-max can be interpreted as probabilities, they need not be the class conditional probability *p*(*a*_*k*_|*x*), even for consistent classifiers. However, the soft-max of a consistent classifier are such that the sets

8 $$  \widehat C_{k} = \left\{ x : q_{\hat h}\left(\left.a_{k}\right|x\right) \geq \max_{j \not = k} q_{\hat h}\left(\left.a_{j}\right|x\right) \right\}  $$

converge, as the sample size increases to infinity, to $A_{k}^{\star }$. However, additional assumptions are needed to ensure that there exists a constant *b*^′^ such that the set

9 $$\begin{array}{@{}rcl@{}}  \widehat D^{\star} & = & \left\{ x : \max_{k} q_{\hat h}\left(\left.a_{k}\right|x\right) > b^{\prime} \right\}, \end{array} $$

asymptotically equals *D*^⋆^ as the sample size tends to infinity. To this end, consider the following definition:

### **Definition 2**

A soft-max estimate is said to be *M*-consistent (or monotone consistent) for the conditional class probability *p*(*a*_*k*_|*x*) if

10 $$ q_{\hat h,n}\left(a_{k},x\right) \longrightarrow_{n \rightarrow \infty} \phi\left(p\left(a_{k},x\right)\right), \quad \forall j,x,  $$

where *ϕ* is a monotone increasing function and the convergence is in probability.

Under that assumption, it is possible to construct, via post-precessing of the soft-max, consistent triage rule from an *M*-consistent soft-max estimator.

### **Theorem 2**

Let $\widehat Y(X)$ is an *M*-consistent soft-max based classifier. Then the associated triage rule defined by the sets (8) and (9) is consistent and converges to the optimal triage rule.

## Example

### A first example

As an illustrative example, we combine deep learning and natural language processing algorithms to classify the primary cancer site, as described by the ICD-O-3 classification manual [3], of 22571 electronic pathology reports from the Louisiana SEER catchment area. The fitted algorithm return a soft-max value for 139 distinct primary cancer site. With minimal optimization, the false positive rate for the classifier is 24.64%, which is significantly higher than the advertised less than 5% classification error from manual classification. This presents us with an opportunity to demonstrate the practical usefulness of triage.

**Fig. 1:**
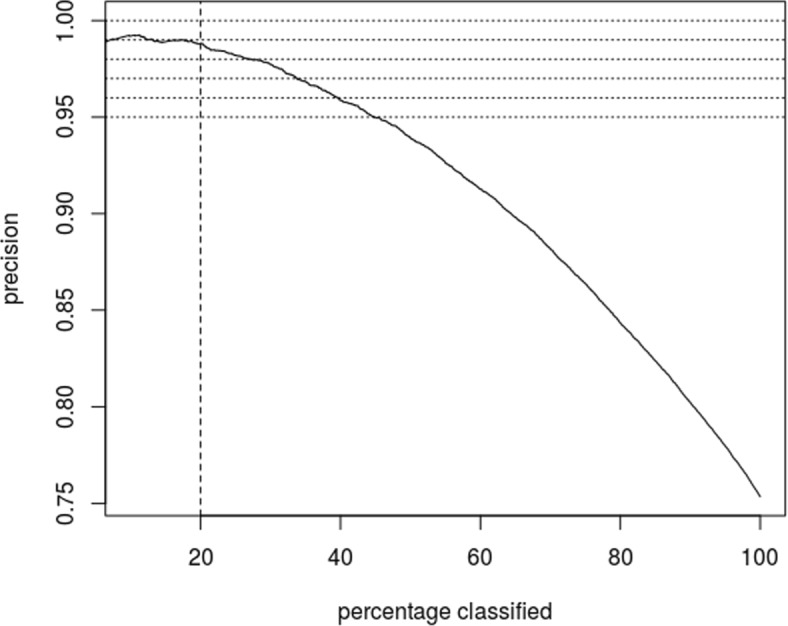
Precision as a function fraction classified. By selectively classifying the 20% of pathology reports having highest max soft-max, one achieves 99% precision. Classifying pathology reports whose max soft-max exceed the median produces a triage rule that has commensurate precision to human classification

Figure 1 graphs the precision as a function of the percentage of pathology reports classified. First note that a precision of 99% can be achieved by selectively classifying about 20% of the pathology reports, and a performance commensurate to manual classification (95%) is achieved by selectively classifying half of the pathology report. Second, we note that the precision is (essentially) a decreasing function of the percentage classified. This implies that the precision is a monotone increasing function of max*jp*(*a*_*j*_|*x*), which is consistent with the *M*-consistency assumption.

### Ad hoc improvement strategy

**Fig. 2:**
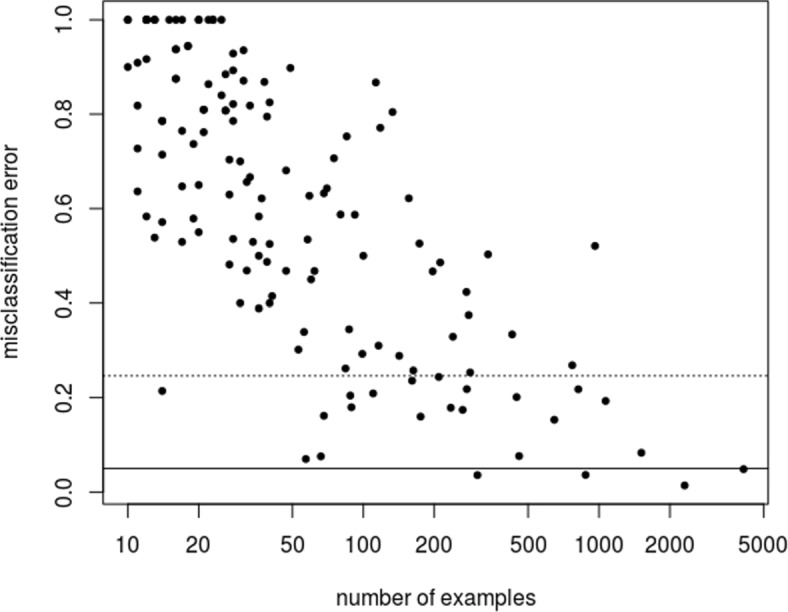
Site specific error rate as a function of number of examples in the dataset. The error rate for the entire dataset is 24.64% and corresponds to the dotted horizontal line

**Fig. 3:**
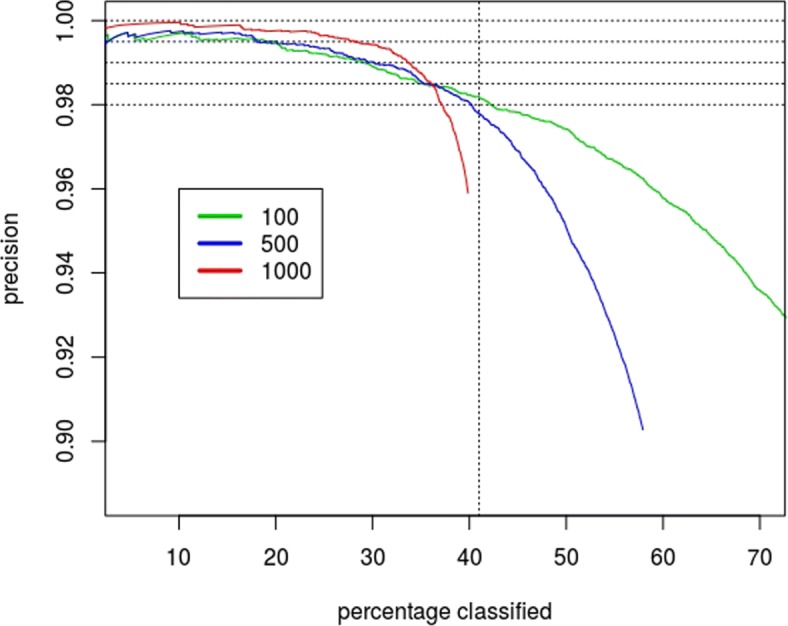
Precision of triage of cancers that have low misclassification rate

The distribution of cancer sites in the registry are not balanced. One might expect that cancer sites with more example are more aptly classified. Examination of Fig. 2, that shows the site-specific error rate as a function of sample size, indicates that this is true in some general sense. This suggests the following ad hoc modification to the triage rules considered so far: decline to classify any cancer site that has not sufficient examples to be well characterized. Figure 3 displays the precision for three choices of sample size: 100, 500 and 1000. The resulting triage rules achieve better than 99% precision on by selectively classifying 30% of the pathology reports, and if we only use the most common cancer sites, sites that have more than a 1000 example, the precision is above 99.5%.

## Conclusion

This paper shows that one can improve a classifiers performance by selectively classifying items, and that the optimal triage rule can be expressed in terms of the Bayes rule. Such triage rule are useful when one seeks machine learning algorithms that achieve a prescribed precision, as is the case in automatic annotation of electronic pathology reports.

## Proofs

### Proof of theorem 1

The misclassification error of a triage rule *T* is

11 $$\begin{array}{@{}rcl@{}} {\mathbb P}[\!T(X) \!\not =\! Y \!\cap \!X \!\in \!D] \!&=& {\mathbb P}[ \!X \in D] - {\mathbb P}[\!T(X) \\[-3pt] &=& Y \cap X \in D] = {\mathbb P}[\! X \in D] \end{array} $$

12 $$\begin{array}{@{}rcl@{}} && - \sum\limits_{k=1}^{m} {\int}_{B_{k} \cap D} p\left(a_{k},x\right) dx. \end{array} $$

For a fixed decision set *D*, the misclassification error is minimized by taking

13 $$  B_{k} = \left\{ x \in D : p\left(a_{k},x\right) \geq p\left(a_{j},x\right), \quad j \not =k \right\}.  $$

To ensure that *B*_*k*_∩*B*_*j*_=*∅* for *k*≠*j*, we apply the convention that if there exists two or more indices for which we have equality, *x* is assigned to set with the lowest index.

To determine the decision set *D* that minimizes the misclassification error subject to the constraint that ${\mathbb P}[X \in D] \geq 1- \varepsilon $, consider all triage rules that satisfy (13)

14 $$\begin{array}{@{}rcl@{}} {\mathbb P}[\!T(X) &&\not = Y \cap X \in D] = {\mathbb P}[\!X \in D] \end{array} $$

15 $$\begin{array}{@{}rcl@{}} \qquad &&- \sum\limits_{k=1}^{m} {\int}_{B_{k}\cap D} p\left(a_{k},x\right) dx \end{array} $$

16 $$\begin{array}{@{}rcl@{}} && = \sum\limits_{k=1}^{m} {\int}_{B_{k} \cap D} f(x) - \max_{j} p\left(a_{j},x\right) dx \end{array} $$

17 $$\begin{array}{@{}rcl@{}} && = {\int}_{D} f(x) - \max_{j} p\left(a_{j},x\right) dx \end{array} $$

Set

$$\psi(x) = f(x) - \max_{j} p\left(a_{j},x\right) $$

and proceed as in the Neyman-Pearson lemma [4] to conclude that optimal decision set *D* is of the form

18 $$ D = \left\{ \frac{\psi(x)}{f(x)} < b \right\},  $$

where the constant *b* is the smallest value for which the constraint ${\mathbb P}[ X \in D] \geq 1-\varepsilon $ is satisfied. The conclusion follows by expressing the ratio

19 $$\begin{array}{@{}rcl@{}} \frac{\psi(x)}{f(x)} &=& 1 - \max_{j} \frac{p\left(a_{j},x\right)}{f(x)} \end{array} $$

20 $$\begin{array}{@{}rcl@{}} &=& 1 - \max_{j} p\left(\left.a_{j}\right|x\right) \end{array} $$
